# Chronic Recurrent Multifocal Osteomyelitis (CRMO): Presentation, Pathogenesis, and Treatment

**DOI:** 10.1007/s11914-017-0405-9

**Published:** 2017-10-27

**Authors:** Sigrun R. Hofmann, Franz Kapplusch, Hermann J. Girschick, Henner Morbach, Jessica Pablik, Polly J. Ferguson, Christian M. Hedrich

**Affiliations:** 1 Pediatric Rheumatology and Immunology, Department of Pediatrics, Medizinische Fakultät Carl Gustav Carus, Technische Universität Dresden, Dresden, Germany; 2 Vivantes Klinikum Friedrichshain, Children’s Hospital, Berlin, Germany; 3 Pediatric Rheumatology and Immunology, Children’s Hospital, University of Würzburg, Würzburg, Germany; 4 Division of Pathology, Medizinische Fakultät Carl Gustav Carus, Technische Universität Dresden, Dresden, Germany; 5 Department of Pediatrics, University of Iowa Stead Family Children’s Hospital, Iowa City, IA USA; 6 Department of Women’s and Children’s Health, Institute of Translational Medicine (Child Health), University of Liverpool, East Prescott Road, Liverpool, L14 5AB UK; 7 Department of Pediatric Rheumatology, Alder Hey Children’s NHS Foundation Trust Hospital, Liverpool, UK

**Keywords:** Chronic non-bacterial osteomyelitis, CNO, Chronic recurrent multifocal osteomyelitis, CRMO, Treatment, Inflammation, Cytokine, Bone, Biomarkers

## Abstract

**Purpose of Review:**

Chronic non-bacterial osteomyelitis (CNO) with its most severe form chronic recurrent multifocal osteomyelitis (CRMO) is an autoinflammatory bone disorder. We summarize the clinical presentation, diagnostic approaches, most recent advances in understanding the pathophysiology, and available treatment options and outcomes in CNO/CRMO.

**Recent Findings:**

Though the exact molecular pathophysiology of CNO/CRMO remains somewhat elusive, it appears likely that variable defects in the TLR4/MAPK/inflammasome signaling cascade result in an imbalance between pro- and anti-inflammatory cytokine expressions in monocytes from CNO/CRMO patients. In this context, we present previously unpublished data on cytokine and chemokine expression in monocytes and tissues.

**Summary:**

CNO/CRMO is an autoinflammatory bone disorder resulting from imbalanced cytokine expression from innate immune cells. Though the exact molecular pathophysiology remains unclear, variable molecular defects appear to result in inflammasome activation and pro-inflammatory cytokine expression in monocytes from CNO/CRMO patients. Recent advances suggest signaling pathways and single molecules as biomarkers for CNO/CRMO as well as future treatment targets.

## Introduction

Chronic non-bacterial osteomyelitis (CNO) is an autoinflammatory bone disorder mostly affecting children and adolescents [1–3]. Autoinflammatory disorders are characterized by an activation of the innate immune system in the absence of high-titer autoantibodies and (at least initially) no involvement of autoreactive lymphocytes. Several genetically inherited monogenic autoinflammatory conditions include early-onset non-infectious osteomyelitis, namely, Majeed syndrome, deficiency of interleukin-1 receptor antagonist (DIRA), and pyogenic arthritis, pyoderma gangrenosum, and acne syndrome (PAPA). Though sharing clinical and pathophysiological features with sporadic CNO, this manuscript will only briefly discuss monogenic autoinflammatory bone disorders and mainly focus on CNO.

**Fig. 1:**
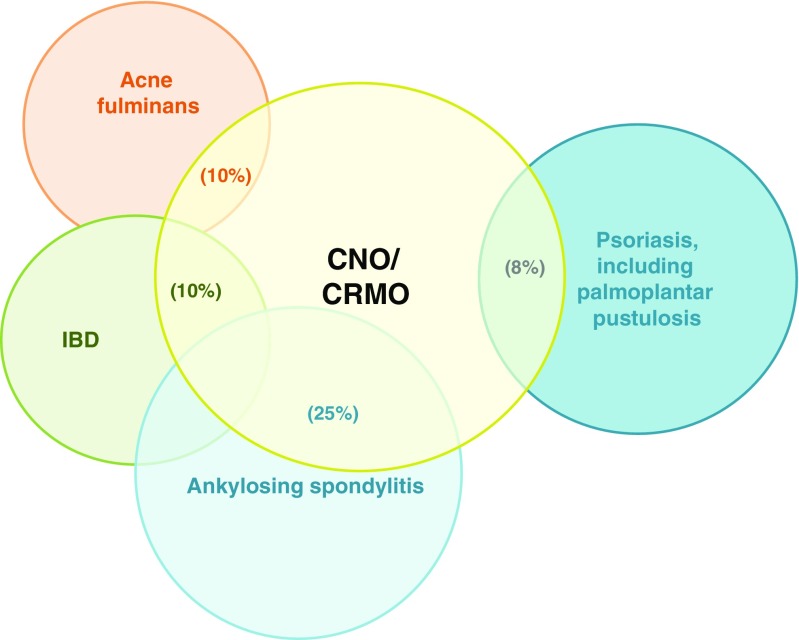
Inflammatory organ involvement in CNO/CRMO. Psoriasis and palmoplantar pustulosis (~ 8%), inflammatory bowel disease (~ 10%), severe acne (~ 10%), and ankylosing spondylitis (~ 25%) have been demonstrated associated with CNO/CRMO [7, 8]. (Figure modified after [8])

Sporadic CNO covers a wide clinical spectrum from rather mild, time-limited, monofocal bone inflammation to severe chronically active or recurrent multifocal bone inflammation. These most severe presentations are referred to as chronic recurrent multifocal osteomyelitis (CRMO). All ethnicities from all geographic regions can be affected. While highest disease incidences appear to exist in Western countries, particularly Central and Northern Europe, it remains unclear whether reporting issues may play a role, since no global epidemiologic studies have been performed. A genetic predisposition for CNO has been suggested by familial clusters of CNO patients [4–6] and associations with other inflammatory conditions, including inflammatory bowel disease, acne, ankylosing spondylitis, and psoriasis (Fig. 1) [1, 2, 7, 9–13]. In adults, patients with sporadic CNO are usually diagnosed with SAPHO, a symptom complex of synovitis, acne, pustulosis, hyperostosis, and osteitis [9, 10]. Thus, SAPHO is currently seen as a closely related disorder with additional symptoms in the adult age group.

## Clinical Presentation and Epidemiology

Clinical presentation and severity of CNO vary significantly between individual patients, covering a wide spectrum with asymptomatic or mild inflammation of single bones at the one end, and chronic recurrent multifocal, and sometimes bone destruction causing osteomyelitis at the other end (then referred to as chronic recurrent multifocal osteomyelitis, CRMO). CNO/CRMO most frequently involves metaphyses of long bones, the pelvic bones, the vertebral column, or the shoulder girdle/clavicle [3, 7, 12].

Clinical signs of bone inflammation include localized skin redness (rare), warmth and/or swelling, and pain. Additional symptoms may be caused by paraosseous inflammation, involving peripheral nerves and/or vessels, skin or bowel inflammation, and synovitis. A subset of CNO patients exhibit inflammatory organ involvement, including psoriasis and palmoplantar pustulosis (~ 8%), inflammatory bowel disease (~ 10%), and severe acne (~ 10%) (Fig. 1) [7]. Some CNO patients develop sacroiliitis, and some patients may progress from childhood CNO to spondylarthropathies in later life stages [14]. In adults, skin inflammation is significantly more common as compared to children. As mentioned above, acne and/or palmoplantar pustulosis frequently occur in the context of synovitis, hyperostosis, and osteitis, which is then referred to as SAPHO syndrome.

Epidemiological data in CNO/CRMO are sparse, and include small case series and regional cohorts. CNO primarily affects children and adolescents, but can generally occur in all age groups. The peak onset of the disease is between 7 and 12 years of age [1–3, 9]. Chronic non-bacterial osteomyelitis is one of the most common autoinflammatory bone disorders in central Europe. According to several case series, CNO may be almost as common as infectious osteomyelitis [7, 15–17]. However, secondary to sometimes rather mild and unspecific clinical symptoms, CNO may be missed.

## Diagnostic Approach

In the absence of widely accepted diagnostic criteria and disease biomarkers, CNO/CRMO remains a diagnosis of exclusion. Clinical signs include bone pain, local swelling, rarely skin redness and heat, associated skin manifestations (including palmoplantar pustulosis, psoriasis, and acne), sometimes mildly elevated temperatures, and pathological fractures (usually of affected vertebral bodies). Non-infectious arthritis can be present in up to 30% of patients [3, 7]. Routine inflammatory parameters (WBC, white blood cell count; CrP, C-reactive protein; ESR, erythrocyte sedimentation rate) are usually normal or mildly elevated.

**Fig. 2:**
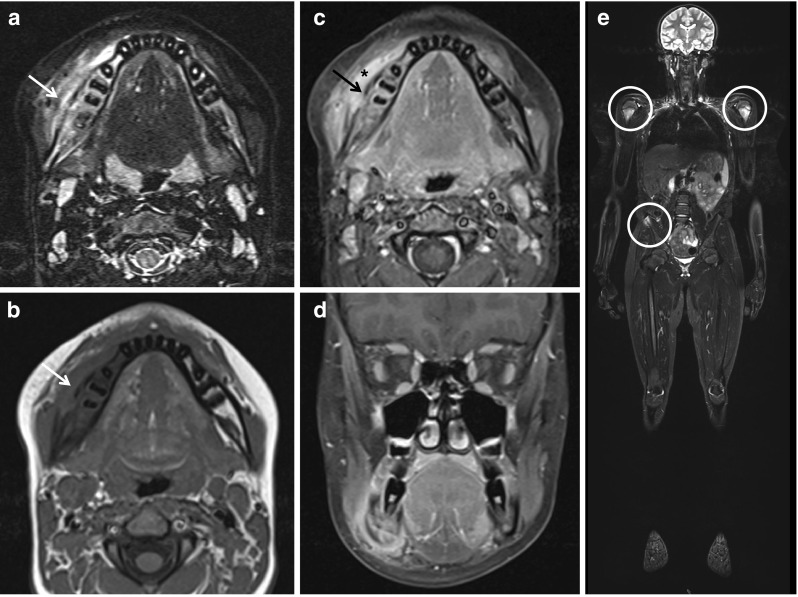
Magnetic resonance imaging in CNO/CRMO. Magnetic resonance imaging in a 15-year-old male patient with swelling and warmth over right mandible. **a** Transversal TIRM sequences unveiled bone swelling and edema of the right mandible (arrow). **b** Native and **c** contrast-enhanced transversal T1 sequences with fat saturation unveiled new bone formation (arrows), resulting swelling, and in **c** enhancement in the right mandible (asterisk). **d** Coronary T1 sequences with fat saturation in the same patient. **e** Whole body MRI (coronary TIRM sequences) unveiled additional sites of bone inflammation at both proximal humeri and the right upper iliac spine (circles) (MRI images with friendly permission from Gabriele Hahn, Pediatric Radiology, Medizinische Fakultät Carl Gustav Carus, Technische Universität Dresden, Dresden, Germany)

Imaging techniques are centrally important for diagnosing CNO/CRMO and excluding differential diagnoses [7]. Inflammatory bone lesions may be detected in plain radiographs as radiolucent, osteolytic, or sclerotic lesions [18–21], but may remain normal in early stages. Particularly in early disease, magnetic resonance imaging (MRI) techniques are highly sensitive. They can detect bone edema even before bone erosions and sclerosis develop, and help assessing inflammation of surrounding tissues. Strongly T2-weighted sequences (Turbo Inversion Recovery Measurement, TIRM) and/or gadolinium-enhanced T1 sequences with fat saturation are used to identify inflammatory bone lesions and/or periosseous affections [21–25]. At the time of diagnosis, whole body imaging using MRI techniques (TIRM) should be performed to identify clinically silent lesions, particularly in the vertebral column [24] (Fig. 2). MRI imaging techniques are also essential for the assessment of disease activity during follow-up, and the identification and monitoring of disease-associated sequelae, which may include fractures, inflammatory involvement and tissue damage to surrounding structures [24].

In unclear cases, bone biopsies are usually performed to exclude chronic infection, malignancies, or other systemic disease. Important differential diagnoses include malignancies (leukemia, lymphoma, primary and secondary bone tumors), infections (bacterial osteomyelitis, tuberculosis, etc.), immunodeficiency (e.g., defects in the IL-12: interferon axis that may be accompanied by mycobacterial infections), Langerhans cell histiocytosis (LCH), and other autoinflammatory disorders (e.g., Majeed syndrome [26–30], DIRA [31], or PAPA [9]). Disease onset before 2 years of age is extremely uncommon and should prompt considering differential diagnoses.

## Mechanisms of Chemokine and Cytokine Dysregulation in CNO/CRMO

Despite recent scientific achievements, the exact molecular pathophysiology of CNO is only incompletely understood. Generally, familial (or monogenic) diseases including CNO as a descriptive symptom can be differentiated from the entity of sporadic CNO, by other disease features. Though not the main focus of this manuscript, studies on monogenic/familial forms of “CNO” contributed to the pathophysiological understanding of sporadic CNO/CRMO.

### Familial/Monogenic CNO

There are at least three human diseases involving chronic multifocal sterile osteomyelitis that are caused by single gene mutations: (i) Majeed syndrome (*LPIN2* mutations), (ii) deficiency of interleukin-1 receptor antagonist (DIRA, mutations in *IL1RN*) [26, 32, 33], and (iii) pyogenic arthritis, pyoderma gangrenosum, and acne syndrome (PAPA, mutations in *PSTPIP1*).

Majeed syndrome is characterized by early-onset multifocal osteomyelitis, dyserythropoietic anemia, and joint contractures. It is caused by loss of function mutations in the *LPIN2* gene, encoding for the lipin 2 protein, a phosphatidate phosphatase (PAP) that plays a role in lipid metabolism. Lipin2-deficient monocytes produce high amounts of pro-inflammatory cytokines IL-6 and TNFα when stimulated by saturated fatty acids. Overexpression of *LPIN2* on the other hand reduces inflammatory cytokine levels [34]. For Majeed syndrome, there is evidence that it is an IL-1β-mediated disease, since bone inflammation and serum inflammation markers improve in response to IL-1β blockade, while TNFα blockers have almost no effect [35].

Deficiency of IL-1 receptor antagonist is characterized by early-onset destructive sterile bone lesions (osteitis and periostitis) and sterile pustulosis of the skin. If left untreated, DIRA leads to a severe systemic inflammatory response syndrome and respiratory failure [32] due to the lack of functional IL-1 receptor antagonist and subsequently uncontrolled IL-1β signaling. Treatment with recombinant IL-1 receptor antagonist results in prompt amelioration and disease control [32, 36].

Pyogenic arthritis, pyoderma gangrenosum, and acne are caused by mutation in the *PSTPIP1* gene, involved in regulation of the actin cytoskeleton. PSTPIP1 binds to pyrin, a central negative regulator of the NLRP3 inflammasome. Therapeutic options for PAPA are local and/or systemic steroids, TNFα blockers, and IL-1 blocking agents [9, 37].

### “Sporadic” CNO/CRMO

Genetic predisposition appears likely to be involved in the pathophysiology of “sporadic” CNO. It was suggested by rare familial clusters of CNO/CRMO and high incidences of comorbid-affiliated inflammatory conditions such as psoriasis and inflammatory bowel disease in CNO patients and first-degree family members (approximately 50%) [7, 13]. Thus, the pathophysiology and inheritance of “sporadic” CNO appears to be complex with, e.g., a combination of associated risk alleles or individually variable (currently unknown) genetic causes resulting in disease resulting in clinically related phenotypes in the absence or presence of environmental factors.

**Fig. 3:**
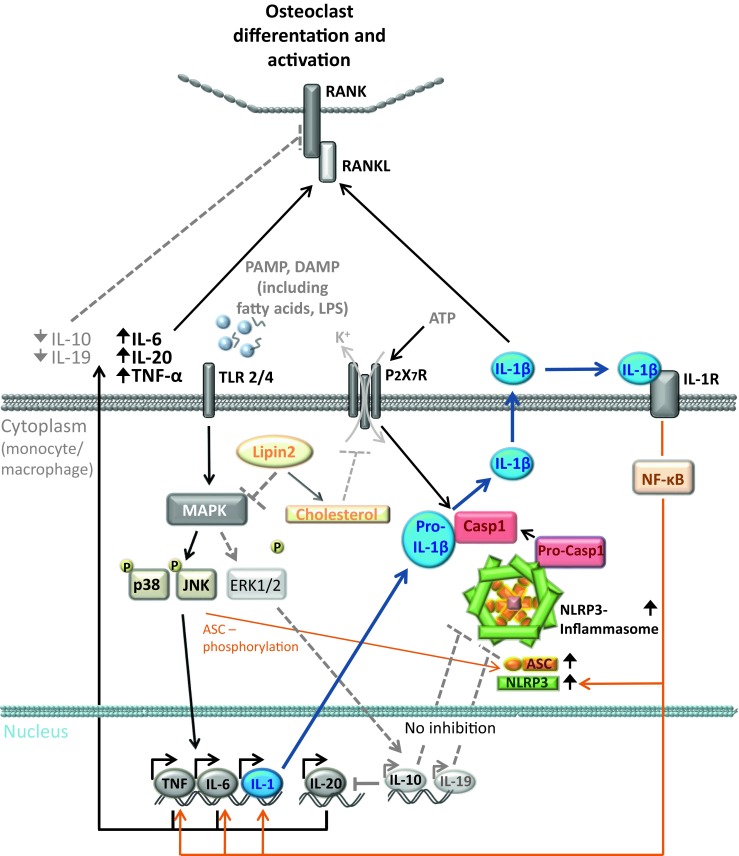
Molecular pathophysiology of CNO/CRMO. Inflammation is a potent and undirected defense mechanism against exogenous pathogens or endogenous danger signals (such as infections, tissue damage). The sensing of danger signals occurs by pattern recognition receptors (PRRs), such as the membrane-associated Toll-like receptors (TLRs) and the predominantly cytoplasmic localized NOD-like receptors (NLRs). After recognition of danger signals by monocytes/macrophages, multiprotein complexes, referred to as inflammasomes are activated. The NLRP3 inflammasome comprises NLRP3, ASC, and procaspase-1. After inflammasome activation, caspase-1 cleaves pro-IL-1β and leads to the secretion of active IL-1β. In monocytes from CRMO patients, MAP kinase Erk1 and 2 signaling is impaired, resulting in reduced expression of the immune regulatory cytokines IL-10 and IL-19. JNK and p38 MAPK are unaffected, leading to the expression of pro-inflammatory cytokines (TNFα, IL-6, IL-1β, IL-20). Reduced expression of IL-10 and IL-19 contributes to increased inflammasome activation and subsequent IL-1β release. Pro-inflammatory cytokines TNFα, IL-6, IL-20, and IL-1β increase the interaction of membrane RANK receptors with their soluble ligand RANKL on osteoclast precursor cells and induce osteoclast differentiation and activation.MAPK: mitogen-activated protein kinase; CRMO: chronic recurrent multifocal osteomyelitis; Erk1: extracellular signal-regulated kinase-1; TLR: Toll-like receptor; IL: interleukin; JNK: Jun kinase; TNF: tumor necrosis factor; NF-κB: nuclear factor-κB; Casp1: caspase-1; PAMP: pathogen-associated molecular pattern; DAMP: danger-associated molecular pattern; RANK: receptor activator of nuclear factor-κB; RANKL: RANK ligand

A tight balance between pro- and anti-inflammatory signals is essential for immune homeostasis. Cytokines and chemokines play key roles in controlling inflammation and instructing immune responses. Consequently, dysregulation of their expression is linked with susceptibility to infectious and autoimmune diseases. It has become increasingly clear that appropriate temporal/spatial expression of cytokines and chemokines may be the key to the delicate balance between inflammation and immunoregulation. The mechanisms that govern the cell type- and receptor-specific induction of cytokines and chemokines, however, in most instances remain unclear [38, 39]. Our current pathophysiological understanding of CNO/CRMO is based on a profound imbalance between pro- and anti-inflammatory cytokines [40, 41••, 42, 43] (Fig. 3). We demonstrated that monocytes from CRMO patients fail to express the immune regulatory IL-10 in response to Toll-like receptor (TLR)4 stimulation with lipopolysaccharide (LPS) [42, 43]. Impaired IL-10 expression is (at least partially) caused by reduced activation of mitogen-activated protein kinases (MAPK), ERK1 and 2 [42], resulting in impaired activation and nuclear shuttling of the transcription factor signaling protein (SP-)1, and subsequently altered recruitment of Sp-1 to the *IL10* promoter. Furthermore, attenuated ERK activation results in reduced histone H3S10 phosphorylation at the *IL10* promotor [42, 43], an activating epigenetic modification. Disturbed epigenetic remodeling and reduced transcription factor binding to the *IL10* promoter result in impaired IL-10 expression and a disruption of balanced pro- and anti-inflammatory cytokine expression.

Together with its homologs *IL19* and *IL20*, the *IL10* gene is organized in the so-called *IL10* cytokine cluster on chromosome 1q32. IL-10 and IL-19 mostly bear immune regulatory functions, while IL-20 exerts pro-inflammatory properties [41••]. Thus, we asked whether the expression of IL-19 and IL-20 are also altered in monocytes from CNO/CRMO patients. In analogy to *IL10*, *IL19* is regulated by Sp-1 and epigenetic remodeling which are altered in monocytes from CRMO patients (H3S10 phosphorylation, DNA methylation), contributing to reduced IL-19 expression in monocytes from CRMO patients [41••].

**Fig. 4:**
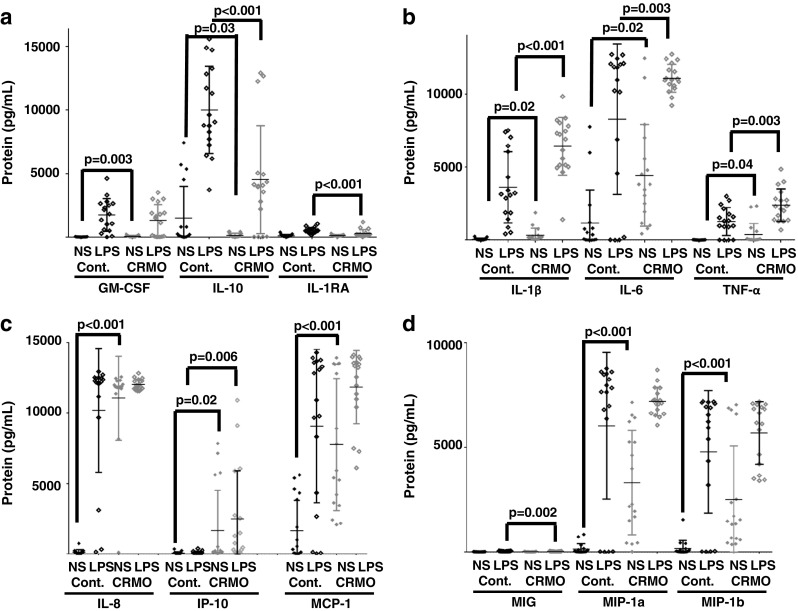
Inflammation marker expression in monocytes from CRMO patients. Monocytes from treatment-naive CRMO patients (*N* = 17) and age and gender matched healthy controls (*N* = 17) were isolated by negative selection of CD14^+^ cells using standard procedures (Miltenyi). Monocytes were cultured at 1 × 10^6^ per milliliter media (RPMI, gentamycine, penicillin) with 5% CO2 at 37 °C. Some cells were stimulated with 100 ng/mL LPS over night as indicated. Protein levels were measured from cell culture supernatants on the LUMINEX2000 platform, using multiplex gene expression arrays (Millipore). **a** Monocytes from CRMO patients fail to express immune regulatory proteins GM-CSF (under resting conditions), IL-10 (under resting conditions and after stimulation with LPS), and IL-1RA (in response to stimulation with LPS). **b** Monocytes from CRMO express increased levels of pro-inflammatory IL-1b, IL-6, and TNFα (under resting conditions and in response to stimulation with LPS). **c** and **d** Monocytes from CRMO patients express increased amounts of pro-inflammatory chemokines IL-8, MCP-1, MIP-1a, and MIP-1b under resting conditions, MIG in response to stimulation with LPS, and IP-10 under resting conditions and in response to stimulation with LPS. These observations further indicate a significant imbalance in the expression of pro- and anti-inflammatory proteins, suggesting a pro-inflammatory phenotype of monocytes in CRMO which show signs of “spontaneous” activation

Notably, the expression of pro-inflammatory IL-20 was not reduced. Contrary, after LPS stimulation, IL-20 expression was enhanced in monocytes from CRMO patients, likely due to reduced DNA methylation of *IL20* [41••]. The expression of other MAPK-induced pro-inflammatory cytokines including TNFα and IL-6 was not reduced but rather increased in monocytes from CRMO patients (Fig. 4b). This may at least partially be explained by the fact that the alternative p38 MAPK pathway was not affected [42].

Recently, inflammatory bone loss and synovial inflammation in IL-10-deficient mice were linked to NLRP3 inflammasome activation [44•]. Furthermore, Scianaro et al. [45] suggested increased NLRP3 inflammasome activation contributing to the inflammatory phenotype in CRMO, showing increased mRNA expression of inflammasome components (ASC, NLRP3, caspase-1) as well as increased IL-1β transcription and release from peripheral blood mononuclear cells from active CRMO patients compared to patients with inactive disease and controls after stimulation with LPS. We recently linked impaired IL-10 and IL-19 expression with increased IL-1β mRNA expression and IL-1β release in monocytes from CRMO patients [41••]. Enhanced inflammasome activation and IL-1β secretion by monocytes from CRMO patients is reversible by co-culture with recombinant IL-10 or IL-19 [41••], suggesting an immunomodulatory function of IL-10 and IL-19 on inflammasome activation.

These observations resulted in the hypothesis that imbalanced expression of anti- (IL-10 and IL-19) and pro-inflammatory cytokines (IL-1, IL-6, TNFα, IL-20) may result in increased osteoclast differentiation and activation through enhanced interaction between receptor activator of nuclear factor-κB (RANK) and its soluble ligand RANKL on osteoclast precursor cells (Fig. 3) [9, 46, 47].

In addition to the molecular mechanisms mentioned above, IL-10 expression is predetermined by genetic variants within the *IL10* proximal promoter region. Three promoter haplotypes rs1800896, rs1800871, and rs1800872, resulting in three haplotype blocks (GCC, ACC, and ATA), influence the capacity of the *IL10* promoter to recruit the transcription factor Sp-1. Interestingly, and to our initial surprise, in cohorts of CRMO patients, *IL10* promoter haplotype blocks encoding for “high” IL-10 expression (GCC) were significantly more common when compared to such encoding for “low” gene expression (ATA) [48]. Provided the molecular pathologic mechanisms discussed above, we hypothesized that individuals with CRMO-associated molecular disturbances and *IL10* promoter haplotype blocks encoding for “low” IL-10 expression may develop more severe disease and may not be diagnosed with CNO/CRMO but other inflammatory conditions. However, this hypothesis remains to be confirmed scientifically.

Recently, a CRMO susceptibility gene has been identified through whole exome sequencing and gene expression microarrays [49]. One homozygous and one compound heterozygous mutation in the filamin-binding domain of the *FBLIM1* gene were detected in unrelated CNO patients from South Asia [49]. Filamin-Binding LIM Protein 1 (FBLIM1) has been suggested to act as an anti-inflammatory molecule that controls bone remodeling through the regulation of RANKL activation via ERK1/2 phosphorylation [49]. On the transcriptional level, FBLIM1 expression is regulated by the transcription factor STAT3 [49]. Since the immune regulatory cytokine IL-10 induces STAT3 activation, aforementioned haplotype blocks within the *IL10* promoter may be involved in the pathophysiology of CNO. Indeed, Cox et al. demonstrated that both individuals carried such *IL10* promoter haplotypes that code for “low” IL-10 expression, which may in turn contribute to reduced STAT3 activation and resulting effects on FBLIM1 expression in the reported individuals [50••].

## Biomarkers for the Diagnosis and Monitoring of CRMO

Currently, widely accepted disease biomarkers for the diagnosis of CNO/CRMO are not available. Recently, we reported a preliminary set of serum inflammatory parameters that allow differentiating between newly diagnosed and treatment naïve patients with CRMO, Crohn’s disease, and healthy controls. Biomarkers included the monocyte derived chemokines monocyte chemotactic protein (MCP-)1 and macrophage inflammatory protein (MIP-)1b, the pro-inflammatory cytokines IL-6 and IL-12, the mast cell derived chemokine eotaxin, RANTES, the soluble IL-2 receptor, and the IL-1 receptor antagonist. Increased serum levels of the inflammatory cytokines IL-6 and IL-12, the chemokines MCP-1, MIP-1b, RANTES, and eotaxin, and the soluble IL-2 receptor distinguished among CRMO patients, individuals with Crohn’s disease and healthy controls. However, the proposed set of biomarkers could not distinguish between patients with CRMO or ANA-positive, HLA B27-negative juvenile idiopathic arthritis (JIA). This may be caused by pathophysiological parallels between the two disorders and/or a currently incomplete set of tested parameters [51]. Currently, follow-up studies are under way, including additional parameters and differential diagnoses.

In addition to serum biomarkers, cytokine and chemokine expression from isolated immune cells may be used to diagnose CNO/CRMO. In Fig. 4, cytokine and chemokine expression patterns from ex vivo isolated peripheral blood monocytes from CRMO patients and controls are provided. Indeed, monocytes from CRMO patients fail to express granulocyte monocyte colony-stimulating factor (GM-CSF), and the anti-inflammatory molecules IL-10 and IL-1 receptor antagonist (IL-1RA) under resting conditions and/or in response to TLR4 stimulation with LPS (Fig. 4a). Conversely, monocytes from CRMO patients express increased amounts of pro-inflammatory cytokines (IL-1β, IL-6, TNFα; Fig. 4b) and chemokines (IL-8, Interferon gamma-induced protein 10: IP-10, MCP-1, MIG, MIP-1a, MIP-1b; Fig. 4c, d) in most cases already under resting conditions. These findings support previous reports on dysbalanced cytokine and chemokine expression and promise potential as disease biomarkers for the diagnosis of CNO/CRMO [41••, 42, 43, 51]. However, additional studies are needed to confirm findings, to extend beyond included differential diagnoses, and to generate longitudinal data sets measuring treatment responses.

**Fig. 5:**
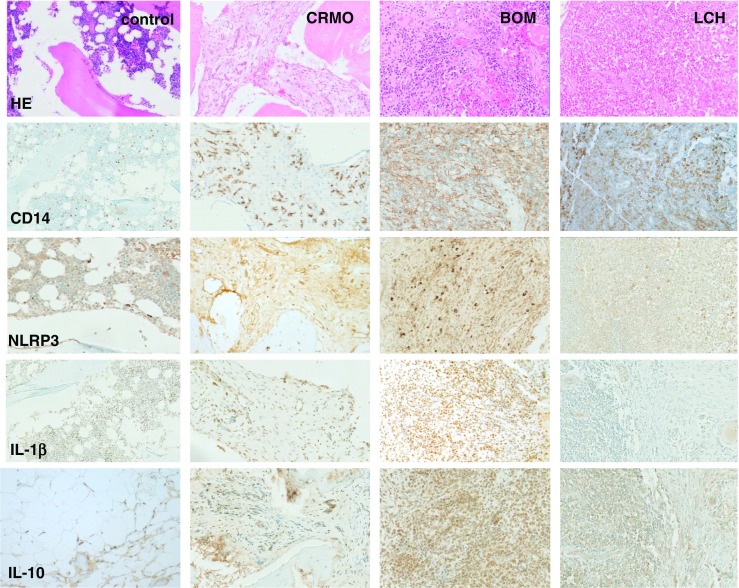
Histomorphological appearance of bone inflammation in CNO/CRMO, bacterial osteomyelitis (BOM), and Langerhans cell histiocytosis (LCH). Formalin-fixed, decalcified, and paraffin-embedded bone biopsy specimens were immune-stained with antibodies directed against CD14 (monocyte marker), NLRP3, IL-1β, and IL-10 using standard techniques (as indicated). Displayed magnification is ×100. In the top panel, HE stains are displayed. Control: trabecular bone with fatty marrow and hematopoietic tissue from a bone healthy patient undergoing osteotomy. CRMO: moderately dense infiltrate of inflammatory cells, predominantly neutrophils and monocytes, some marrow fibrosis. BOM: dense infiltrate of inflammatory cells with predominant neutrophils, cellular bone remodeling and bone necrosis. LCH: Ovoid Langerhans cells, some with linear grooves of nuclei, admixed with inflammatory cells, including a large number of eosinophils, lymphocytes, neutrophils, and plasma cells. Infiltrates of CD14-positive monocytes are a central component of inflammation in acute BOM, early phase CRMO, and LCH. Expression of the inflammasome component NLRP3 is increased in inflammatory infiltrates of BOM > CRMO > LOM, translating into IL-1 β protein expression in BOM and CRMO. As suggested by studies in ex vivo isolated monocytes, IL-10 expression in inflammatory bone lesions from CRMO patients is reduced as compared to lesions from BOM and LCH patients

Lastly, gene expression profiles in situ may be used as disease biomarkers. Preliminary studies in bone biopsies from patients with CRMO, Langerhans cell histiocytosis (LHC), bacterial osteomyelitis (BOM), or from healthy controls delivered promising results. In agreement with the aforementioned observations in ex vivo isolated monocytes, IL-10 expression in bone tissue from CRMO patients appears reduced when compared to other inflammatory conditions (LHC and BOM; Fig. 5). Expression of the inflammasome component NLRP3 and the pro-inflammatory cytokine IL-1β is increased in samples from CRMO patients when compared to LHC patients or healthy controls. Patients with BOM, however, (not surprisingly) exhibit even higher inflammasome activation and IL-1β expression levels when compared to CRMO (Fig. 5).

## Murine Models of CRMO

Studies in primary human cells and in cell lines are limited by intrinsic and/or extrinsic factors, including sample sizes, availability (in primary human cells), culturing conditions, and biological limitations in immortalized cells. Several murine models are available to study underlying pathologic mechanisms of non-infectious bone inflammation. Mice deficient of proline-serine-threonine phosphatase-interacting protein 2 spontaneously develop bone inflammation, elevated pro-inflammatory cytokines in the blood, extramedullary hematopoiesis and skin inflammation, resembling very severe CRMO (Table 1).

**Table 1:** *Pstpip2* mutant mice strains as disease models for CRMO. *cmo* chronic multifocal osteomyelitis, *ENU* N-ethyl-N-nitrosourea, *Pstpip2* proline-serine-threonine phosphatase-interacting protein 2

*Pstpip2* mutant mice	Generation	Mutation	References
*lupo* mice	ENU mutagenesis, homozygous mutation	p.I282N c.Y180C	[52, 53]
*cmo* mice	Homozygous spontaneous mutation	p.L98P Exon5: c.T293C	[26, 54, 28, 55, 56]
Targeted knockout	Conventional knockout Conditional knockout	Target exons 3 and 4	[57]

*Lupo* mice carry a chemically induced homozygous mutation (c.Y180C; p.I282N) in the proline-serine-threonine phosphatase-interacting protein 2 (*Pstpip2*) gene [52, 53]. Chronic multifocal osteomyelitis (*cmo*) mice carry a spontaneously acquired homozygous mutation (c.T293C, p.L98P) in *Pstpip2*. To date, the exact molecular contribution of *Pstpip2* mutations to sterile bone inflammation remains somewhat unclear [57]. Pstpip2 belongs to the F-BAR (Fes/CIP4 homology-Bin/Amphiphysin/Rvs) domain containing protein superfamily, which couples membrane remodeling with actin dynamics associated to endocytic pathways and filopodium formation [58]. It is a cytosolic, cytoskeleton-associated adapter molecule, which interacts with formin binding protein 17 (FBP17) through its F-BAR domain. In the presence of Pstpip2, an antagonistic recruitment of FBP17 and Pstpip2 to the plasma membrane enables correct activation of actin polymerization at podosomes. In the absence of Pstpip2, actin polymerization is hyperactivated by constitutive membrane recruitment of the FBP17-WASP (Wiskott-Aldrich syndrome protein) complex [59]. Macrophages that express Pstpip2 at reduced levels exhibit abnormal podosome formation, leading to a more invasive phenotype.

Interleukin-1β has been linked with the pathogenesis of osteomyelitis in *cmo* mice [60, 61••]. Cmo mice lacking the functional IL-1 receptor I (IL-1RI) or IL-1β (but not IL-1α) were completely protected from CNO [60, 61••]. Conversely, *cmo* mice deficient of the inflammasome components NLRP3, ASC or caspase-1 developed severe CNO, indicating that there must be another kinase or protease other than caspase-1-activating IL-1β. Previously described proteases, performing IL-1β cleavage includes neutrophil serine proteases or caspase-8 [62••], suggesting that neutrophils may play a central role in disease pathogenesis. Thus, Cassel et al. performed experiments to identify immune cell subsets critical for IL-1β release in *cmo* mice [60]. LPS-primed and ATP-stimulated bone marrow cells but not bone marrow derived macrophages from *cmo* mice produced high amounts of IL-1β. Increased IL-1β production was reduced by treatment of *cmo* bone marrow with a serine protease inhibitor (diisopropylfluorophosphate) but not with the pan-caspase-1 inhibitor z-YVAD-fmk [60], suggesting the involvement of neutrophils in the pathogenesis of *cmo*. Findings were confirmed by Lukens et al. [61••], who additionally showed that pharmacological depletion of neutrophils with the monoclonal antibody anti-Ly6G protected *cmo* mice from CNO [63•]. Interestingly, *cmo* mice either deficient of caspase-1 or -8 developed CNO, whereas *cmo* mice deficient of both caspases were protected from disease [63•], indicating that both caspases play redundant roles in *cmo* mice.

Taken together, in agreement with aforementioned findings in monocytes from CRMO patients, recent work in *cmo* mice suggests a central involvement of IL-1β in disease pathophysiology. Complete deficiency of Pstpip2 results in dysregulated production of IL-1β by neutrophils and enhanced osteoclastogenesis. Of note, myeloid cells determine the phenotype in Pstpip2-deficient animals (at least initially) independent of the adaptive immune system [54].

Over the recent years, it became increasingly clear that host interactions with skin and gut microbiota have significant effects on immune homeostasis [64]. Particularly in genetically predisposed individuals, alterations to microbiomes can result in uncontrolled inflammation and the expression of autoimmune/inflammatory conditions. Recent data from Lukens et al. [63•] suggest that dietary manipulation of the microbiome in *cmo* mice can prevent osteomyelitis. The exact molecular mechanisms involved, however, remain unclear. Patients with CRMO exhibit associations with severe acne (10%) and inflammatory bowel disease (10%) [7], which underscores the therapeutic potential of these observations, since all of these CRMO-associated disorders are characterized by significant alterations to microbiomes [64].

## Treatment and Monitoring

**Fig. 6:**
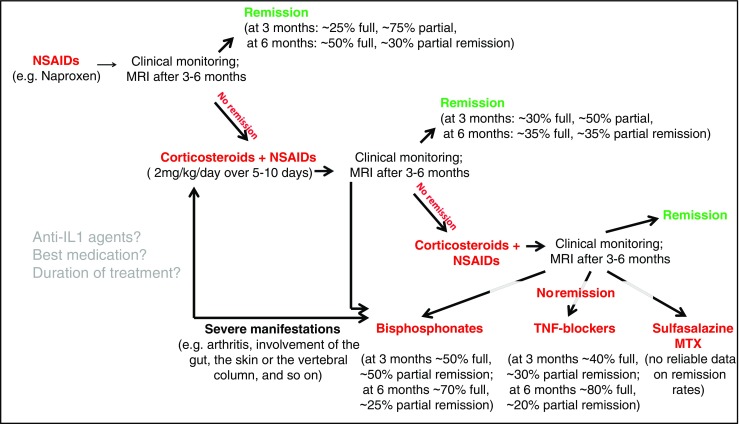
Treatment of CNO/CRMO. NSAIDs (e.g., naproxen) are usually applied as first-line therapy in CNO/CRMO patients. Monitoring includes clinical investigations and MRI scans after 3 to 6 months. Treatment goals are clinical and, in the case of vertebral involvement, radiological remission. In NSAID-refractory cases, treatment can be escalated with corticosteroids. The authors usually apply 2 mg/kg oral prednisone per day plus NSAIDs over 5 to 10 days. In cases who first respond to corticosteroid treatment but then flare, high-dose steroids (2 mg/kg/day) can be repeated and supplemented by low-dose corticosteroids (0.1–0.2 mg/kg/day) over a longer period, e.g., to “bridge” until DMARDs are working. In individuals who fail to reach clinical and (if vertebrae are involved) radiological remission or relapse again, bisphosphonates, TNFα inhibitors, sulfasalazine, or methotrexate (MTX) should be considered. In patients with vertebral body involvement and structural damage, aggressive treatment should be discussed initially, e.g., with bisphosphonates

Treatment of patients with CNO/CRMO is largely based on expert opinion and relatively small case collections. It usually involves non-steroidal anti-inflammatory drugs (NSAIDs), corticosteroids, disease-modifying anti-rheumatic drugs (DMARDs, usually methotrexate or sulfasalazine), anti-TNF agents, or bisphosphonates (Fig. 6).

Several retrospective analyses and one prospective observation indicate that NSAIDS are effective in a large subset of patients within the first 1–2 years of treatment. We, however, recently documented that more than 50% of patients flare after 2 years [7, 65•]. Corticosteroids appear to quickly and effectively control inflammatory activity, but rarely induce long-term remission. Though only reported in a small number of patients, treatment with pamidronate or anti-TNF agents has been reported to be highly effective in a significant percentage of CNO/CRMO patients, inducing long-lasting remission in a large subset of patients [3, 9, 66]. However, all reports are limited by retrospective data collection and relatively small patient numbers.

Large prospective clinical trials to determine the best medication and the duration of treatment are lacking. Currently, consensus treatment plan initiatives of the North American Childhood Arthritis and Rheumatology Research Alliance (CARRA) and the German Society of Pediatric Rheumatology (GKJR) are under way and will investigate and compare treatment responses to currently used therapeutic agents and protocols.

In addition to the currently applied treatment options, blockade of other molecules may be beneficial in CNO/CRMO. Since IL-1β is involved in the molecular pathophysiology of CNO/CRMO, recombinant IL-1 receptor antagonist anakinra or anti-IL-1 antibody treatment with canakinumab may be applied. Furthermore, IL-1 blockade proved effective in familial monogenic autoinflammatory disorders involving CNO (namely, Majeed syndrome and DIRA) [32, 35]. However, surprisingly few cases of anti-IL-1 treatment have been reported and showed mixed response with variable outcomes. The potential explanation for the poor response may include low tissue concentrations, pathophysiological heterogeneity in CNO/CRMO, among others.

Blockade of RANKL with the recombinant RANK ligand inhibitor denosumab may reduce osteoclast activation and inflammatory bone loss in CNO/CRMO and promises potential in the treatment of CNO/CRMO. However, at this point, there are no reliable reports on successful application of these treatment options in CNO/CRMO.

Monitoring disease activity is a concern in CNO/CRMO patients, since a significant subset of patients may develop pain amplification syndrome. Thus, clinical scores including pain scores, routine inflammatory parameters, and imaging results have been suggested (PedCNO score) [66]. However, to date, scores are incompletely evaluated and involve time consuming and costly investigations (e.g., MRI). Thus, easily accessible and inexpensive disease biomarkers for the assessment of disease activity are urgently warranted. We recently reported a preliminary set of biomarkers (IL-12, MCP-1, sIL-2R) that may act as markers for treatment response to NSAIDs [51]. Though included as a parameter in PedCNO scores, ESR and CrP correlated less closely with PedCNO scores when compared to the reported serum biomarkers. Thus, after further evaluation in independent cohorts and in response to other treatment options, IL-12, MCP-1, and/or sIL-2R may be used as future biomarkers for disease activity in CNO/CRMO.

## Conclusions

Chronic non-bacterial osteomyelitis with its most severe form chronic recurrent multifocal osteomyelitis is an inflammatory bone disorder that can result in damage to bones and other tissues. Due to the lack of widely accepted diagnostic criteria or disease biomarkers, CNO/CRMO remains a diagnosis of exclusion. The molecular pathophysiology of CNO/CRMO remains incompletely understood. However, dysregulated cytokine expression from innate immune cells centrally contributes to the inflammatory phenotype of CNO. Treatment is largely based on small, mostly retrospective case collections and expert opinion, and prospective studies are largely lacking. Currently, consensus treatment protocols are under way and will deliver reliable data on treatment responses and outcomes. The recent identification of several genetic and molecular alterations in CNO/CRMO promise future success in determining exact pathophysiological causes and target-directed treatment options.
